# Unusual cystic presentation of pheochromocytoma in a case of suspected von Hippel–Lindau disease

**DOI:** 10.1016/j.radcr.2026.06.005

**Published:** 2026-06-29

**Authors:** Shambhavi Bisht, Gaurav Raj, Arpit Agarwal

**Affiliations:** Dr. RMLIMS, Lucknow, India

**Keywords:** Von Hippel–Lindau disease, Cystic pheochromocytoma, Adrenal lesion, Pancreatic cysts, Computed tomography

## Abstract

Von Hippel–Lindau (VHL) disease is a rare autosomal dominant disorder characterized by multisystem involvement. Pheochromocytoma is a known manifestation of VHL and typically presents as a solid hypervascular adrenal mass. Cystic pheochromocytoma is an uncommon variant and may pose a diagnostic challenge, especially in the absence of classical clinical features. We report the case of a 51-year-old male who presented with acute pancreatitis. Contrast-enhanced computed tomography revealed multiple cystic lesions involving the pancreas, kidneys, and liver along with a predominantly cystic lesion replacing the left adrenal gland showing a small enhancing solid component. In the background of suspected VHL disease, the adrenal lesion was considered likely to represent a cystic pheochromocytoma. This case highlights the importance of recognizing atypical imaging appearances of pheochromocytoma and considering an underlying syndromic association such as VHL in the presence of multisystem involvement. Early identification is crucial for appropriate management, surveillance, and screening of family members.

## Introduction

Von Hippel–Lindau (VHL) disease is an autosomal dominant condition caused by mutations in the VHL gene on chromosome 3p [1]. The global prevalence of VHL is estimated to range from 1 in 36,000 to 1 in 91,000 individuals [2].

VHL is characterized by the development of multiple benign and malignant lesions across various organs. The most common tumors include retinal and central nervous system hemangioblastomas, renal cell carcinoma, pheochromocytoma, pancreatic neuroendocrine tumors, and endolymphatic sac tumors [3]. The incidence of pheochromocytoma in VHL is approximately 10%-20%, with most tumors being solid or mixed solid cystic in morphology [4].

We present a case of suspected VHL highlighting the rare occurrence of a predominantly cystic pheochromocytoma, emphasizing its atypical imaging features and diagnostic challenges (Fig. 1, Fig. 2).

**Fig. 1:**
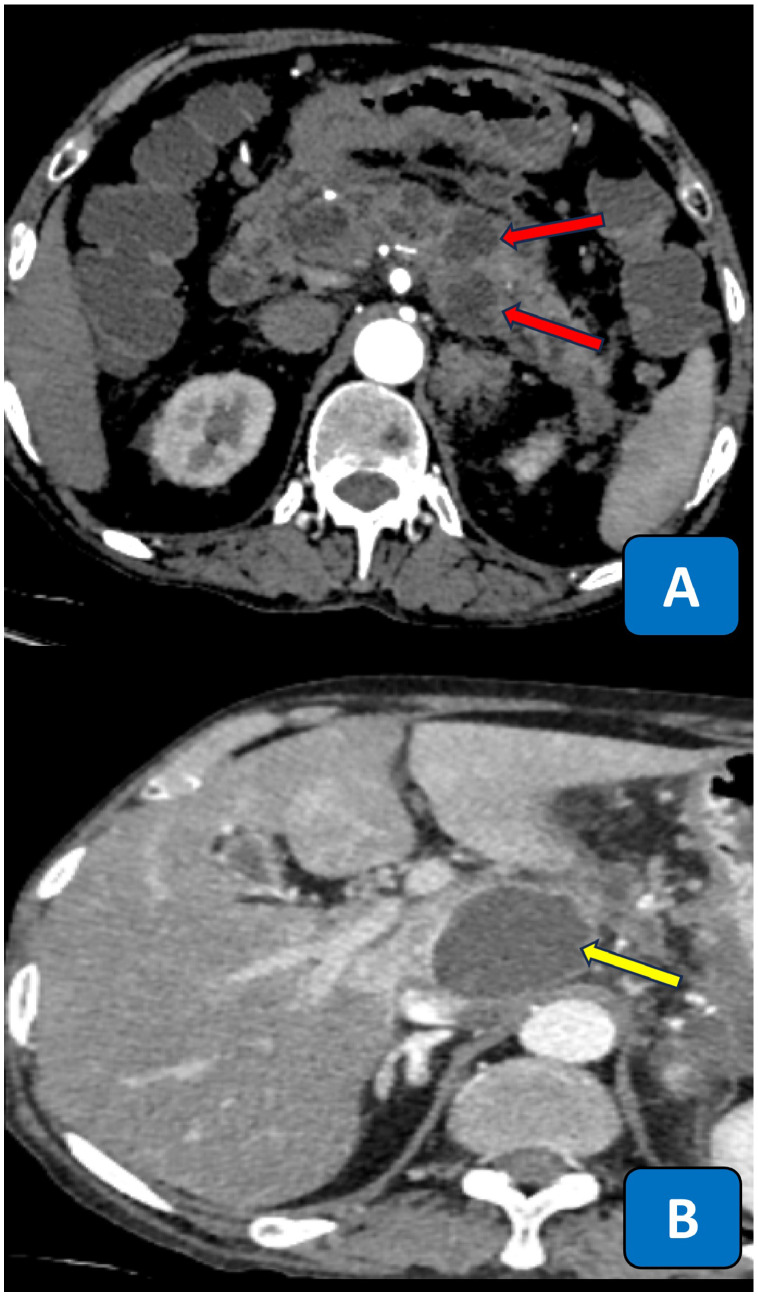
Contrast-enhanced CT of the abdomen. Axial section (A) demonstrates multiple variable-sized cystic lesions within the pancreatic parenchyma (red arrows). Axial section (B) demonstrates a large cystic lesion in the caudate lobe of the liver (yellow arrow).

**Fig. 2:**
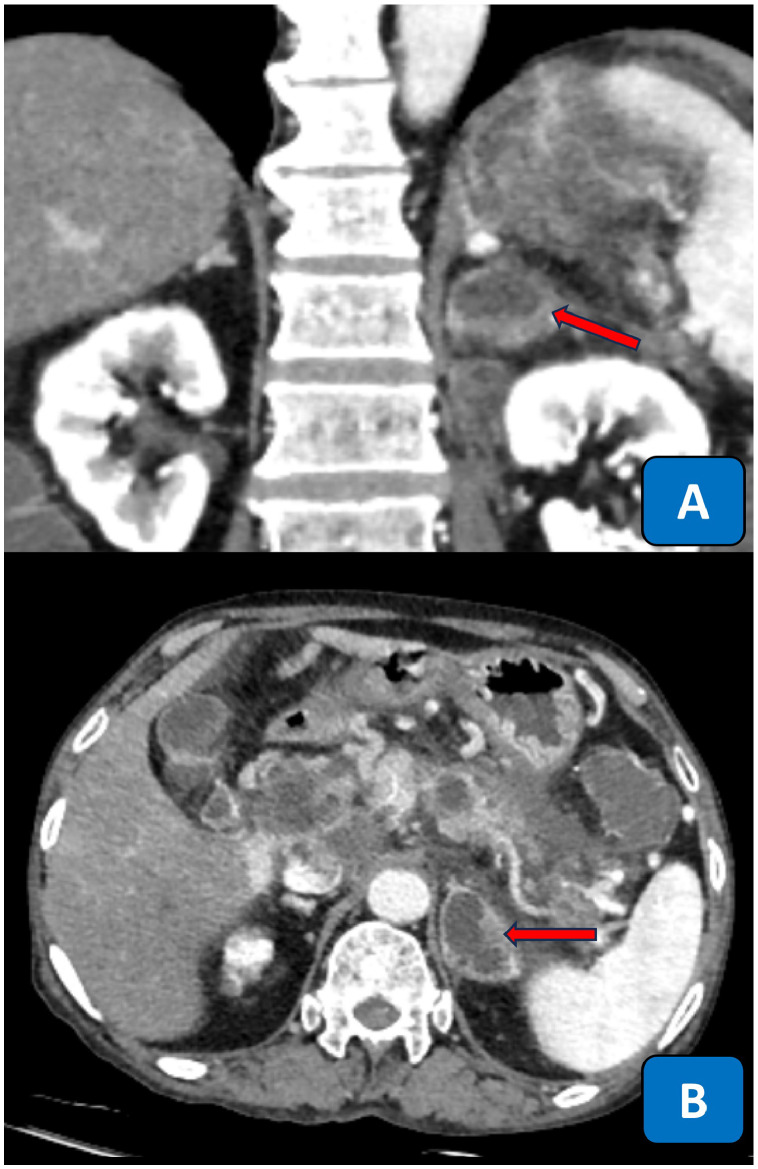
Contrast-enhanced CT of the abdomen. Coronal (A) and axial (B) sections demonstrate a well-defined cystic lesion in the left suprarenal region (red arrows), replacing the left adrenal gland, with a small enhancing solid component. Associated fluid is noted in the peripancreatic region and the lesser sac.

## Case summary

A 51-year-old male presented with complaints of abdominal pain for the past 1 month, which had progressively increased in severity and was not relieved by medications. The pain was associated with nausea and vomiting. He was a known case of hypertension for 1 year and diabetes mellitus for 5 years with a history of occasional alcohol intake over the past 20 years. The patient also reported episodic headache, palpitations, and weight loss.

Routine blood investigations revealed elevated serum amylase (∼725 U/L) and serum lipase (∼583 U/L), along with raised total leukocyte count (∼19.9 × 10³/µL). A diagnosis of acute pancreatitis was made, and the patient was referred to the radiology department for contrast-enhanced CT of the abdomen to assess for complications of pancreatitis.

Contrast-enhanced CT of the abdomen performed on a 64-slice scanner revealed multiple cystic lesions in the liver, pancreas, and bilateral kidneys. Imaging revealed near-complete replacement of pancreatic parenchyma with multiple variable-sized cystic lesions. The entire left adrenal gland was replaced by a multiloculated cystic lesion with a small enhancing solid component. In addition, features of pancreatitis were present, including peripancreatic, perirenal, and bilateral paracolic gutter fat stranding with a few loculated collections in the lesser sac and perisplenic region. Mild prominence of the pancreatic duct was noted without evidence of pancreatic calcification.

A provisional radiological diagnosis of acute-on-chronic pancreatitis with walled-off necrosis was made in the background of suspected VHL, with an associated mixed solid-cystic left adrenal lesion, likely representing a cystic pheochromocytoma.

Biochemical evaluation was performed and revealed mildly elevated plasma-free normetanephrine levels (526.9 pg/mL) with normal plasma-free metanephrine levels.

Owing to its atypical predominantly cystic appearance and the need for definitive tissue diagnosis, CT-guided biopsy of the left adrenal lesion was performed under appropriate hemodynamic precautions, with emergency support and interventional radiology backup readily available.

Histopathological examination revealed nests (zellballen pattern) of polygonal tumor cells with abundant eosinophilic granular cytoplasm separated by delicate fibrovascular stroma. Areas of cystic degeneration and hemorrhage were identified, correlating with the imaging appearance. Immunohistochemistry showed tumor cells positive for chromogranin and synaptophysin, confirming the diagnosis of cystic pheochromocytoma.

## Discussion

Pheochromocytoma is an uncommon tumor of the adrenal medulla arising from chromaffin cells [5].

Pancreatic involvement in Von Hippel–Lindau disease is frequent, with a prevalence as high as 87.4% [6]. It typically manifests as simple cysts and serous cystadenomas and rarely as pancreatic neuroendocrine tumors [7]. Multiple renal cysts are common in VHL and may act as precursors to clear cell renal cell carcinoma [8]. Pheochromocytoma occurs in approximately 10%-20% of patients with VHL, making it the second most common tumor associated with the disease [9].

On imaging pheochromocytomas typically appear as solid hypervascular masses arising from the adrenal gland. Predominantly cystic pheochromocytomas are rare and may result from intratumoral hemorrhage, necrosis, or degeneration [10]. Such atypical presentations can pose diagnostic challenges, especially in patients without classical symptoms of catecholamine excess [11]. In the present case the lesion was predominantly cystic with only a small enhancing solid component, making differentiation from other adrenal cystic lesions difficult. Differential diagnoses for predominantly cystic adrenal lesions include adrenal cyst, adrenal pseudocyst, cystic metastasis, and necrotic adrenal neoplasms. The possibility of pheochromocytoma was considered due to suspected VHL disease.

The pancreatic cystic lesions in the present case were considered likely VHL-related pancreatic cysts/serous cystic lesions. However, the pancreatitis was considered more likely related to the patient’s chronic alcohol intake rather than the pancreatic cystic lesions themselves.

Pheochromocytomas associated with VHL are typically identified at a younger age and are often clinically silent [12]. In this case, the diagnosis at an advanced age is atypical and may indicate delayed recognition.

The diagnosis of pheochromocytoma is established through biochemical testing with plasma free metanephrines or urinary fractionated metanephrines when imaging findings are atypical [13]. Timely diagnosis is important, as unrecognized tumors carry a risk of severe hypertensive episodes, especially during operative procedures [14].

The combination of pancreatic cysts, renal cysts, hepatic cysts, and an adrenal lesion in this patient is highly suggestive of VHL syndrome. Identifying this pattern is essential to enable appropriate follow-up, genetic evaluation, and screening of family members due to its autosomal dominant inheritance [1,15].

There was no contributory family history. Retinal examination/fundoscopy was unremarkable. MRI screening of the brain and spine as well as genetic testing for VHL mutation could not be completed as the patient declined further investigations and treatment, which represents a limitation in the present case.

Management of pheochromocytoma includes preoperative α-adrenergic blockade followed by surgical excision. Lifelong biochemical and imaging surveillance is recommended in syndromic cases because of the risk of recurrent and multifocal tumors [5].

## Conclusion

This case demonstrates a rare cystic presentation of pheochromocytoma in the setting of suspected Von Hippel Lindau disease. The presence of multiple cystic lesions across different organs should raise suspicion for an underlying syndromic association such as VHL. Recognizing these features is important for guiding further evaluation, confirming the diagnosis, and initiating surveillance and screening of family members.

## Patient consent

Written informed consent was obtained from the patient for the publication of his medical data and radiological images.
